# Design and Synthesis of Chitosan—Gelatin Hybrid Hydrogels for 3D Printable *in vitro* Models

**DOI:** 10.3389/fchem.2020.00524

**Published:** 2020-07-14

**Authors:** Sofia Magli, Giulia Beatrice Rossi, Giulia Risi, Sabrina Bertini, Cesare Cosentino, Luca Crippa, Elisa Ballarini, Guido Cavaletti, Laura Piazza, Elisa Masseroni, Francesco Nicotra, Laura Russo

**Affiliations:** ^1^Department of Biotechnology and Biosciences, University of Milano-Bicocca, Milan, Italy; ^2^G. Ronzoni Institute for Chemical and Biochemical Research, Milan, Italy; ^3^Department of Medical and Surgical Science, University of Milano-Bicocca, Milan, Italy; ^4^Department of Environmental Science and Policy (ESP), University of Milan, Milan, Italy

**Keywords:** glycopolymers, hybrid hydrogels, functionalization strategies, Diels-Alder click reaction, 3D bioprinting, 3D cultures, click chemistry for 3D cellular models

## Abstract

The development of 3D printable hydrogels based on the crosslinking between chitosan and gelatin is proposed. Chitosan and gelatin were both functionalized with methyl furan groups. Chemical modification was performed by reductive amination with methyl furfural involving the lysine residues of gelatin and the amino groups of chitosan to generate hydrogels with tailored properties. The methyl furan residues present in both polymers were exploited for efficient crosslinking via Diels-Alder ligation with PEG-Star-maleimide under cell-compatible conditions. The obtained chitosan-gelatin hybrid was employed to formulate hydrogels and 3D printable biopolymers and its processability and biocompatibility were preliminarily investigated.

## Introduction

3D cultures embedded in hydrogels represent a challenging opportunity to advance in tissue engineering and 3D *in vitro* functional models (Ashammakhi et al., 2019). The advent of new technologies, such as 3D printing and bioprinting, allows the production of artificial 3D cell microenvironments, provided that a wide range of printable hydrogels are available (Moroni et al., 2018; Bagher et al., 2019). Such hydrogels must be biocompatible and able to provide 3D scaffolds with the appropriate structural and chemical features, such as stiffness, viscosity, and capacity to interact with cells providing them with the required biological signals to address their fate (Ooi et al., 2017; Neves et al., 2019). New fascinating strategies have been developed to better control the encapsulation of one or more cell lines in specific architectures, all based on the use of proper biomaterials with tailored properties and fabrication strategies. An important issue is, therefore, the availability of biomaterials suitable for different therapeutic purposes and fabrication strategies. Most or commercially available biopolymers employed as matrices for cell cultures are not suitable or adaptable as bioinks for 3D printing protocols, and vice versa. 3D printing protocols are strongly related to the physical properties of the polymers employed. At the same time, cells embedded in the printable polymers need motifs and functional groups able to mimic biochemical signals and structures present in the natural extracellular matrix (Nicolas et al., 2020). The development of new accurately functionalized biopolymers that enable the properties of the final constructs to be tuned is therefore desirable. The majority of the biopolymers employed for biomedical use are natural polymers extracted from animal tissues or obtained by recombinant methods. They are biocompatible and suitable for integration in biological systems, but their applications are often limited due to the poor mechanical properties, the inadequate architecture, and the limited modularity of the structural features. In order to overcome these limitations, one of the most promising strategies is based on the combination of polymers by controlled crosslinking with linkers of different lengths, in order to “tune on demand” the morphological and mechanical properties of the final constructs (Spicer et al., 2018). Chitosan is a cationic polysaccharide characterized by N-acetyl-D-glucosamine and D-glucosamine as units. Chitosan derivatives have been already shown to recreate a microenvironment conducive to cell growth (Zhang et al., 2015) and they have been extensively employed for tissue engineering applications (Polgar et al., 2017; Fasolino et al., 2019; Ruprai et al., 2019; Sultankulov et al., 2019; Cassimjee et al., 2020; Tao et al., 2020). Moreover, chitosan can be combined with natural polymers such as gelatin, which contains specific aminoacidic residues such as Arg-Gly-Asp (RGD) in its sequence (Davidenko et al., 2016). This amino acid sequence is present ubiquitously as an adhesion sequence in the proteins of extracellular matrix (Liu et al., 2004) and is involved in numerous physiological functions. Binding between integrins and RGD induces a series of reactions in the cytoplasm involving the cytoskeleton and other proteins that regulate cell adhesion, growth, and migration. For this reason, the combination of these two polymers has been widely investigated for various biomedical applications from wound healing (Huang et al., 2013; Carvalho, 2017) or drug delivery (Kim et al., 2018). Chitosan-Gelatin hybrids have been identified as promising hybrid materials for tissue engineering or drug delivery applications (Afewerki et al., 2019; Rodríguez-Rodríguez et al., 2020). The use of hybrids obtained by ionic interactions or covalent linkages has been investigated to obtain scaffolds or hydrogels with specific kinetics and degradation properties (Gorgieva and Kokol, 2012). Different fabrication methodologies have been employed depending on the final intended application, such as crosslinking by chemical reaction of complementary groups using glutaraldehyde (Jiankang et al., 2009) or N, N-(3-dimethylaminopropyl)-N′-ethyl carbodiimide (Alizadeh et al., 2013) as crosslinkers or crosslinking by high-energy irradiation like UV (Saraiva et al., 2015; Carvalho, 2017). However, conventional crosslinking methods involve the use of toxic reagents such as glutaraldehyde or photoinitiators and mutagenic UV irradiation and lead to the formation of side-products that can be unsafe or not fully biocompatible. In the present work, we are presenting an alternative based on Diels-Alder click chemistry that is applicable to different formulation and fabrication strategies at physiological pH without further purifications, also allowing cell encapsulation during the crosslinking without affecting cell viability.

This strategy requires the introduction in the biopolymer chains of functional groups able to react with sufficiently fast kinetics in mild and biocompatible conditions and without the formation of toxic side products—in other words, a “click reaction” (Nimmo and Shoichet, 2011; Azagarsamy and Anseth, 2013; Tam et al., 2017). A linker with complementary functional groups is commonly used for click reaction crosslinking. Several biocompatible click reactions have been employed to obtain smart biomaterials and to impart them with new biological functionalities (Nimmo and Shoichet, 2011; Russo et al., 2011, 2014, 2016; Azagarsamy and Anseth, 2013; Lin et al., 2013; Gandavarapu et al., 2014; Nair et al., 2014; Taraballi et al., 2014; Huynh et al., 2018; Kaur et al., 2018). However, the application of these procedures on heterogeneous systems is of growing interest in materials science also for the improvement of bioprinting hybrid polymer procedures.

In order to fulfill this objective, we have investigated a strategy to functionalize gelatin and chitosan, selected as biopolymers, in order to obtain a final construct with both polysaccharide and protein properties. According to our experience, the most reproducible crosslinking approach is the Diels-Alder reaction (Roy et al., 2015). The Diels-Alder cycloaddition has already been employed to generate polysaccharide-based biomaterials, as with hyaluronic acid and alginate-based hydrogels, employed to encapsulate cancer cell lines, confirming the biocompatibility of the produced biomaterials (Smith et al., 2018).

**Scheme 1:**
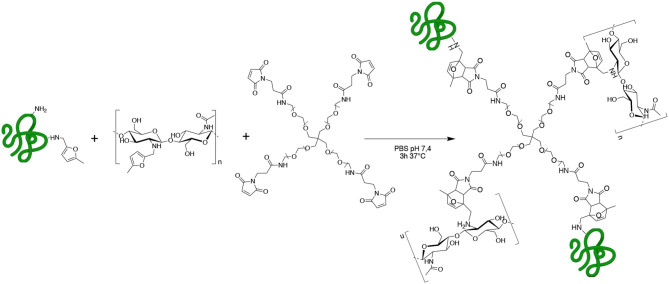
GE-CH crosslinked network obtained by methyl furan-maleimide Diels–Alder (DA) click reaction.

In the present work, we designed and studied gelatin (GE) and chitosan (CH) functionalization with methyl furfural as a diene and the employment of the functionalized constructs as starting polymers for the design of a customizable hybrid biomaterial crosslinked by Diels-Alder cycloaddition with a commercial Star-PEG functionalized with maleimide groups as dienophile (Star-PEG-MA) (Scheme 1).

Chitosan and gelatin were treated with 5-methyl furfural in the presence of NaCNBH_3_ to perform a reductive amination, taking advantage of their amino groups, to generate the methyl furan functionalized biopolymers. This reaction has already been investigated on single-chain polymers for the generation of new diagnostic and therapeutic tools for nanomedicine and tissue engineering applications (Hall et al., 2011; Nimmo et al., 2011; Alge et al., 2013; Gandini, 2013; Koehler et al., 2013a,b; Park et al., 2014; Gregoritza and Brandl, 2015; Stewart et al., 2016; Ma et al., 2017; Tam et al., 2017; Smith et al., 2018; Madl and Heilshorn, 2019).

However, to our knowledge, the employment of Diels-Alder crosslinking to produce hybrid systems based on protein and polysaccharide components functionalized with methyl furan moieties has not been investigated yet. As a matter of fact, the crosslinking reaction between two totally different biopolymers containing the same reactive functional group must be accurately modulated to generate a hybrid material with the required properties. The intensity of derivatization of GE and CH with methyl furan was therefore determinated in order to finally obtain the most efficient crosslinking conditions.

The hydrogel network formation was assessed using different concentrations of maleimide tetra-functionalized PEG (Star-PEG-MA commercially available) to select the most promising formulation. In detail, the methyl furan-functionalized biopolymers were mixed and reacted with star-PEG-MA to obtain the final crosslinked hydrogel (GE-CH). The different reactivities of gelatin and chitosan with the Star-PEG-MA make it problematic to assess the appropriate degree of functionalization for obtaining network formation in the crosslinking step. On the other hand, the Diels-Alder cycloaddition turned out to be an affordable method to control the crosslinking of the final hydrogel and to easily quantify the degree of functionalization of both of the polymeric components by NMR.

**Figure 1:**
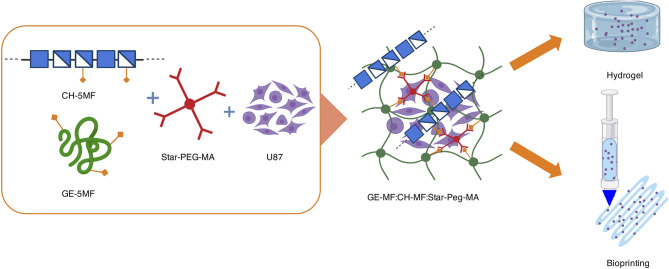
Graphical representation of hybrid formation, cell encapsulation, and formulation in a cell-laden hydrogel bioprinted construct.

The hydrogels obtained were manufactured by employing different formulation strategies (Figure 1) and were preliminary assessed for different biomedical applications. We tested the hybrid hydrogels for spheroid encapsulation studies, in which the applicability of commercial materials is often limited in terms of histological analysis feasibility and reproducibility. Furthermore, the biomaterial of choice must avoid uncontrolled migration or low viability of the embedded cells.

We also screened our hybrid materials as biopolymers for 3D-bioprinting applications, generating cell-laden constructs. 3D bioprinting is today an emerging fabrication technology with potential applications in tissue engineering and cell biology studies (Gungor-Ozkerim et al., 2018; Sun et al., 2020). However, also in this case, libraries of bioprintable biomaterials need to be created to enable more effective *in vitro* testing and to overcome the current limitations arising from the different cell population requirements and the multitude of physiological and pathological conditions to mimic.

## Materials and Methods

Gelatin (type A), 5-methylfurfural, 4arm-PEG10K-Maleimide (Star-PEG-MA), Phosphate-Buffered Saline, U87 glioblastoma cell line, Eagle's minimal essential medium, L-glutamine, Sodium Pyruvate, Fetal Bovine Serum, penicillin, and streptomycin were purchased from Sigma-Aldrich, Italy. Water-soluble chitosan was purchased from Carbosynth Ltd, UK. A LIVE/DEAD Cell Viability Assay was purchased from ThermoFisher.

### Functionalization of Methyl-Furan Functionalized Gelatin (GE-MF)

Gelatin type A (2.00 g) was dissolved in 30 ml of PBS at pH 4.5 and heated at 37°C until a homogeneous solution was obtained. To the dissolved gelatin, 6.8 ml of 5-methyl furfural was added and left under gentle stirring. After 30 min, 2.15 g of NaBH_3_CN was added, followed by stirring for 3 h. The solution was dialyzed against a NaCl solution (0.1 M) for 1 day, followed by mQ H_2_O for 4 days, using 14 kD dialysis membranes at 40°C. Functionalized polymers were purified through filtration, using 0.5 and 0.22 mm filters. The obtained solution was freeze-dried to give 1.73 g of a white spongy solid.

### Functionalization of Methyl-Furan Functionalized Chitosan (CH-MF)

Chitosan (2.00 g) was dissolved in 35 ml of 2% acetic acid solution and mixed by sonication and vortex until a homogeneous solution was obtained. To the dissolved chitosan, 206 μl of 5-methyl furfural was added, and it was left under gentle stirring. After 30 min, 65 mg of NaBH_3_CN were added, and the reaction was stirred for 3 h at room temperature. The solution was dialyzed against 0.01 M NaCl solution for 1 day, followed by mQ H_2_O for 4 days, using 1 kDa dialysis membranes at 40°C. Functionalized polymers were purified through filtration using 0.5 and 0.22 mm filters. The obtained solution was freeze-dried to give 1.37 g of a white spongy solid.

### Hybrid Hydrogel and Dried Sample Formation

#### Hydrogel Network

GE-MF (33 mg) and CH-MF (17 mg) were dissolved in 0.750 ml of PBS at 7.4 pH by vortexing at 37°C until complete dissolution. PEG-Star-MA (2.5 mg) was dissolved in 0.250 ml at rt, added to the hybrid solution, and mixed. The hybrid solution (GE-CH) was left for 3 h at 37°C to allow for hydrogel network formation.

#### Dried Samples for SEM and Swelling Studies

the GE-MF, CH-MF, and PEG-Star-MA solution formed as previously described was transferred into Teflon® molds (15 cm diameter) and left for 3 h at 37°C. Once the Diels-Alder reaction had occurred, the sample was transferred at −20°C for 24 h and then freeze-dried for 48 h to obtain cylindrical samples (Irmak et al., 2019). Scanning Electron Microscopy (SEM) was employed to characterize the cross-section of the fibrous dried samples obtained.

### H-NMR

Spectra of chitosan and its derivate were obtained with a Bruker AVANCE III HD 500 MHz spectrometer (Bruker, Karlsruhe, Germany) equipped with a 5-mm TCI cryogenic probe at 303 K. Spectra were processed with BrukerTopspin software version 4.0.6. For preparation, 60 mg of chitosan sample was solubilized in 10 ml aqueous acid solution (Acetic acid 2%) and was mixed for 24 h at room temperature. Then, 1 ml of solution was lyophilized and solubilized in 0.6 ml D_2_O. The ^1^H NMR spectrum was acquired with presaturation of residual HDO, using 64 scans, an 8-s relaxation delay, and 32 k time-domain points. Spectra of gelatin and its derivate were also obtained with the Bruker AVANCE III HD 500 MHz spectrometer (Bruker, Karlsruhe, Germany) equipped with a 5-mm TCI cryogenic probe at 333 K and processed with BrukerTopspin software version 4.0.6. Samples of about 6 mg were dissolved in 0.6 ml of D_2_O. The ^1^H NMR spectra were acquired with presaturation of residual HDO, using 128 scans, a 25-s relaxation delay, and 32 k time-domain points.

### FT-IR

All of the FT-IR spectra were recorded in attenuated total reflection ATR mode using a PerkinElmer Spectrum 100 FTIR Spectrometer. All of the samples had been coated onto a steel surface and were analyzed at different points of the material. The absorbances of the samples and backgrounds were measured using 25 scans each. The spectral absorption data were collected in the range between 4,000 and 650 cm^−1^ at a spectral resolution of 2 cm^−1^.

### SEM Analysis

Scanning Electron Microscopy (SEM) was employed to characterize the surface and the cross-section of the obtained fibrous samples. The morphology of the final hybrid biomaterials was characterized using a ZEISS Gemini 500 field emission HR-SEM at voltage of 5 kV. Prior to examination under SEM, all of the samples were sputter-coated with a 10-nm chrome layer.

### Swelling Analysis

The swelling analysis was performed according to the literature (Varaprasad et al., 2011). In summary, the dried GE-CH crosslinked polymers were employed as dried cylinders (5 mm height and 15 mm diameter) and fully immersed in 10 ml of pH 7.4 PBS at 37°C. Samples were collected at the indicated time points, and the weights of the samples were measured using an electronic balance. The followed equation was employed to calculate the swelling ratio:

(1) $$\begin{array}{l} \text{Swelling }\%=\frac{\text{Ws }-\text{ Wd}}{\text{Wd}}\times 100 \end{array}$$

$$\begin{array}{l} \left[\text{Wd}=\text{Weight of polymer};\text{ Ws}=\text{weight of swollen polymer}\right] \end{array}$$

### Rheological Properties

The rheological properties of the hydrogel were studied using a CMT rheometer (DHR-2, TA Instruments, USA) equipped with a 40-mm-diameter plate–plate geometry. For all tests, the temperature and the gap between the plates were kept constant 37°C and 1.0 mm, respectively, and a solvent trap was used to prevent loss of solvent. The viscoelastic behavior of the material at the mesoscale was investigated by means of dynamic measurements and quantified through the storage modulus [or elastic component of the complex modulus G^*^(ω)] G’(ω), and the loss modulus [or viscous component of the complex modulus G^*^(ω)] G”(ω) [Pa]. G’(ω) and G”(ω) characterize the solid-like and fluid-like contributions to the measured stress response that follows a sinusoidal deformation of the tested material, respectively. The range of linear viscoelastic response under oscillatory shear conditions was identified by means of a strain sweep test: the sample was subjected to an extended field of strains (0.01–100%) at a constant frequency of 1 Hz. The mechanical plots were then drawn by performing a frequency sweep test over the 0.01–100 rad/s frequencies at a constant strain (2%). Finally, a step strain sweep test was carried out to investigate the self-healing properties of the sample in response to applied shear forces. Viscoelastic properties were measured as a function of time in an oscillatory time sweep (3 min, 2% strain, 1 Hz frequency) before and after severe destruction of the gel network (800% strain, 3 min, 1 Hz frequency). The extent of the self-healing behavior was calculated according to Zhao et al. (2014) (Equation 1) as the ratio of the storage moduli of the healed (${\text{G}}_{\text{h}}^{\prime }$) and pristine gels (${\text{G}}_{\text{p}}^{\prime }$).

(2) $$\begin{array}{c} \text{Healing Efficienty }(\text{HE})={\text{G}}^{\prime }\text{h}/{\text{G}}^{\prime }\text{p} \end{array}$$

Data were analyzed with TRIOS 3.0.2 software.

### Cell Culture

Before bioprinting and use to create spheroid structures, human glioma U87-MG cells were maintained in adhesion condition in T75 tissue culture flasks. U87 cells were cultured in Dulbecco's modified Eagle's medium supplemented with 10% fetal bovine serum, 100 units/ml penicillin, and 100 mg/ml streptomycin at 37°C under a humidified atmosphere with 5% CO_2_.

### 3D Bioprinting Procedure and Biocompatibility

GE-MF (66 mg) and CH-MF (34 mg) were dissolved in 1.5 ml of PBS at 37°C and vortexed until complete dissolution. PEG-Star-MA (5 mg) was dissolved in 0.5 ml of PBS at room temperature, added to the GE-CH hybrid solution, and mixed. The GE-CH solution was left for 30 min under UV-light for further sterilization and 2 h at 37°C to obtain partial network formation of the hydrogel solution. U87 glioblastoma cells (7 × 10^5^/ml) in complete medium were added to the GE-CH solution (5%, 2 ml) and transferred into a 5-ml bioprinter syringe (Moroni et al., 2018). Each sample was bioprinted as a grid on 35-mm Petri TC dishes using a 22G nozzle with a 0.41 mm diameter at 25–35 KPa. After printing, cells were maintained at 37°C with 5% CO_2_. The culture media were refreshed every 2 days. The viability of the cells exposed to the bioprinting conditions was evaluated using a LIVE/DEAD viability/cytotoxicity kit (Invitrogen®). Stock solutions of the assay, ethidium homodimer-1 (0.036 μM), and calcein-AM (1 μM), were prepared in PBS. A volume of 1 mL calcein stock solution was added to each bioprinted sample. Following 20 min of incubation at 37°C, 1 ml of ethidium homodimer-1 stock solution was added to the sample, and then it was incubated for an additional 10 min at 37°C (Ooi et al., 2018). The stained bioprinted models were washed three times with PBS before obtaining images. Imaging analysis was performed with a CELENA® S Digital Imaging System with a TC PlanAchro 4X Ph objective. Cell viability was calculated as (number of green-stained cells/number of total cells) × 100 using Fiji ImageJ software (Schindelin et al., 2012).

### 3D Spheroid Formation and Histological Analysis

To form spheroids, U87 cells were seeded 5 × 10^3^ per well in 100 μl of culture medium into 96-well round-bottom ultra-low attachment plates (Corning) and incubated for 5 days. Spheroids were deposited in GE-CH hydrogel using a 24-well plate.

In order to fix hydrogel-embedded spheroids and to obtain a compact hydrogel structure, 10% buffered formalin was added for 2 h at RT into the well. After fixation, hydrogel-embedded spheroids were washed in PBS and were moved into histological cassettes, adding filter paper pieces on top and bottom of the sample to avoid loss of material. Samples were paraffin-embedded with a tissue processor (ETP, Histo-Line Laboratories) using a standard protocol, cross-sectioned at 3-μm thickness by rotary microtome (Leica RM2265), mounted on glass slides, and stained with Haematoxylin and Eosin (H&E). Sample sections were observed under a light microscope (Olympus BX51). Representative images were captured with a digital camera (Evolution VF digital Camera) using Image-Pro Plus software.

### Statistical Analysis

Results are presented as mean ± *SD* and compared using one-way ANOVA. Statistical significance was set at *p* < 0.05.

## Results and Discussion

**Scheme 2:**
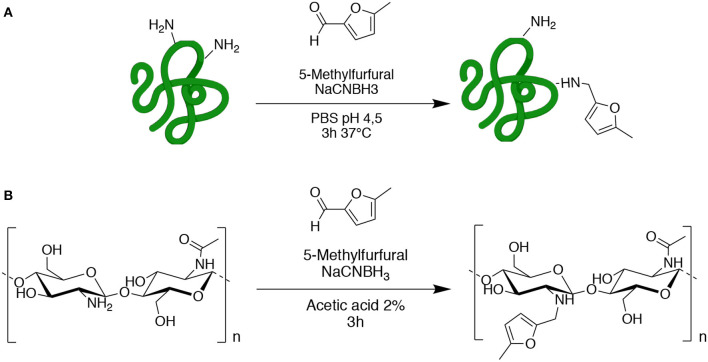
Functionalization of gelatin **(A)** and chitosan **(B)** with 5-methylfurfural by reductive amination.

Gelatin (GE) and chitosan (CH) were chosen as commercially available starting materials with biocompatible properties. Both GE and CH starting polymers were functionalized by reductive amination with 5-methyl furfural, in order to obtain the methyl-furan derivatives GE-MF and CH-MF (Scheme 2). CH-MF and GE-MF were characterized by chemical-physical methods to determine the reproducibility of the reaction and the degree of functionalization. With these aims, FT-IR and NMR analyses were performed, taking advantage of the fact that methyl furan is an “unnatural group” normally absent in natural proteins and polysaccharides, and therefore, it can be easily detected, and the degree of functionalization was dosed in the obtained products.

**Figure 2:**
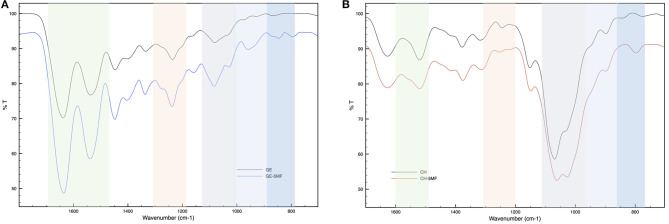
FT-IR spectra of **(A)** functionalized and unfunctionalized GE **(B)** functionalized and unfunctionalized CH.

The FT-IR spectrum of GE-MF was compared with that of the untreated gelatin as control. As shown in Figure 2A, the spectrum of untreated gelatin shows in the green region the characteristic two peaks at 1,635 and 1,535 cm^−1^ corresponding to C=O and -NH- of the amide II, respectively. In the case of GE-MF, in the blue-scale region, the signals of C=C, C-H, and -C-O-C- corresponding to the furan ring are detectable, respectively, at 843, 786, and 1,080 cm^−1^. CH and CH-5MF were also analyzed by FT-IR and showed the peaks at 1,625, 1,520, and 1,315 cm^−1^ corresponding to C=O stretching (amide I), NH bending (amide II), and C-N stretching (amide III) of amide groups due to the partial acetylation (Figure 2B) (Wang et al., 2016). As for the gelatin spectrum, also in this case in the blue-scale zone, the signals of C=C, C-H, and -C-O-C- of the furan are detectable between 800 and 1,100 cm^1^.

To confirm the functionalization and to determine the degree of functionalization, NMR spectra were registered, and comparisons of the untreated and functionalized biopolymers were performed.

**Figure 3:**
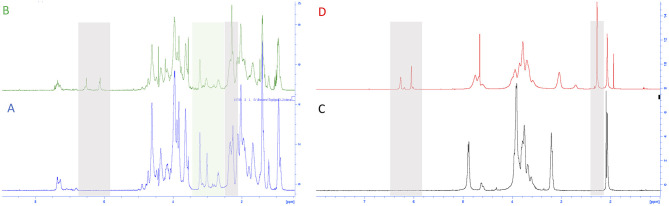
^1^H NMR spectra of **(A)** gelatin control (GE), **(B)** functionalized gelatin (GE-5MF), **(C)** chitosan control, and **(D)** functionalized chitosan (CH-5MF).

We analyzed gelatin and functionalized gelatin by ^1^H NMR to verify the derivatization of lysine amino groups. In methyl furan derivate (Figure 3B), the lysine signal at 2.9 ppm decreases compared to the intensity of the same peak in the gelatin spectrum (Figure 3A). Moreover, new signals (in gray) attributed to the methyl furan structure are exhibited at 6.4, 6.2, and 2.4 ppm (Nimmo et al., 2011; Koehler et al., 2013c). The degree of functionalization was calculated as previously reported in the literature, showing a degree of substitution of 14% (Hoch et al., 2013).

The properties and features of chitosan, such as solubility and biodegradability, are related to its degree of acetylation (DA). NMR spectroscopy is one of the most accurate methods for determining the DA for chitosan (Fernandez-Megia et al., 2005). Figures 3C,D shows ^1^H NMR spectra, respectively, of CH and CH-5MF. Since the chitosan solution is generally viscous, its NMR spectrum has been recorded at 333 K. Various expressions were worked out to calculate the degree of acetylation; we integrated the peaks related to the acetyl group, comparing anomeric protons and found that the DA value is 18% (Figure S1). Also, new signals attributed to the methyl furan structure at 6.4, 6.2, and 2.4 ppm are presented in the spectrum. The functionalization degree has been calculated as reported in the literature to be 18% of substitution.

### 3D Network Formation by Diels-Alder Reaction

**Figure 4:**
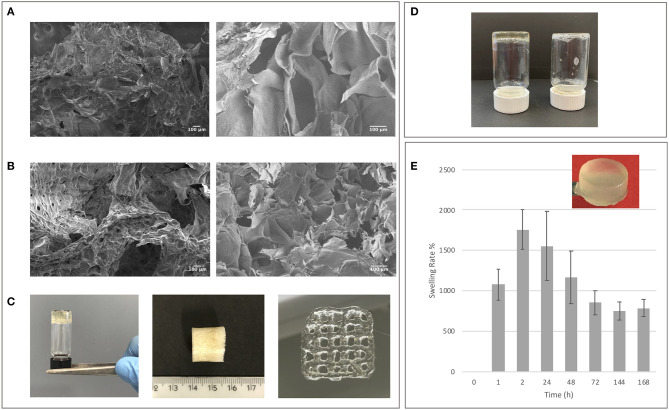
**(A)** SEM control images of non-crosslinked GE-CH samples. **(B)** SEM images of crosslinked GE-CH samples. **(C)** GE-CH hydrogel, dried samples, and printed formulations. **(D)** Test tube inversion method confirming hybrid hydrogel formation of GE-CH compared with unfunctionalized GE-CH in liquid form. **(E)** Swelling analysis of GE-CH hydrogel reported at time points from 0 to 168 h.

Star-PEG-MA (10.000 MW) with four arms was selected to allow substantial spatial freedom in the network formation to favor cell viability during the 3D bio-printing process but also to better control the reactivity of functional groups during Diels-Alder cycloaddition (Smith et al., 2018). The obtained GE-MF and CH-MF were employed for biomaterial network formation using the Star-PEG-MA, as showed in Scheme 1. Different amounts of Star-PEG-MA were employed in order to determine the best kinetics of network formation for the final crosslinked hydrogel. The hybrid hydrogel with a % m/V ratio of CH-GE:PEG-Star-MA=1:3:0.05 and a final concentration of 5% in PBS 7.4 was selected due to the optimal properties in terms of stability and viscosity of the final hydrogel network. The selected GE-CH hybrid hydrogel was then characterized and tested to formulate both the hydrogel and the bioprintable hydrogel. The formation of crosslinked GE-CH hydrogel was studied in comparison with the unfunctionalized GE and CH polymers in the presence of Star-PEG-MA by test tube (Figure 4D). The GE-CH hybrid was produced in hydrogel form and preliminary printed without cells. The produced hybrid was employed to assess the processability of the produced hybrid network and to characterize its swelling behavior and morphological properties (Figure 4C). SEM microscopy of the final construct shows homogeneous structures with interconnected pores, as shown at higher magnification (Figures 4A,B). The swelling ability of hydrogel is important for subsequent *in vitro* cell studies and to develop biomaterial-based cellular constructs. The swelling is also connected to the ability to absorb nutrients from the microenvironment and to favor cell adhesion. The swelling studies were performed at pH 7.4 and 37°C to characterize both the stability and the water uptake of the produced biomaterials. The final hybrid biomaterial shows the greatest swelling rate (1,700%) between 2 and 24 h; however, by 72 h, the swelling decreases to 800% in the absence of polymer degradation and release into the medium. These results could be related to the free functional groups of the hybrid material resulting in a different structural organization of polymer chains during water uptake (Mao et al., 2006; Saraiva et al., 2015; Li et al., 2017; Guaresti et al., 2019). The hybrid reaches swelling equilibrium at 144 h, showing adequate properties for *in vitro* cell studies. Concerning potential final applications like tissue engineering or 3D bioprinted cell models, the biomaterials should have a slow degradation rate. As shown in Figure 4E, the crosslinking methodology efficiently maintained the integrity of the hybrid hydrogel at 37°C in PBS 7.4, demonstrating the effect of the covalent linkage in the control of biomaterial degradation over time until 45 days (data not show).

### Rheological Analysis

**Figure 5:**
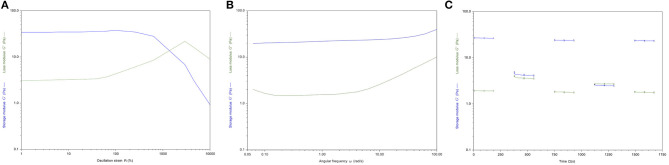
**(A)** Storage (G′, blue) and loss (G″, green) moduli vs. oscillation strain (γ) for GE-CH. **(B)** Storage (G′, blue) and loss (G″, green) moduli vs. angular frequency (ω) for GE-CH hydrogel. **(C)** Structural recovery behavior of the GE-CH as a function of time, assessed by monitoring G′ (t) (γ2%, ω1 rad/s) after destruction by applying an 800% oscillatory shear strain. Modulus G′ (blue) and G” (green).

The rheological analysis was carried out on homogenous hydrogel solutions prepared as described in the Material and Methods section. The strain sweep test (Figure 5A) showed a linear viscoelasticity zone (LVE), where the intrinsic structural properties of the samples are independent of the applied stress and where the storage modulus (G′) is higher than the loss modulus (G″). In the terminal LVE, the deformation is so large that a liquid-like behavior prevails; that is, the yield point is reached. The crossover points of the dynamic moduli were calculated. GE-CH hydrogel showed a linear strain region up to about 40% (Figure 5A). The 2% strain value was then selected for subsequent sweeps. The crossover point occurred at a very high value of strain. This value of LVE is typical of entanglement networks and strong gels (Ross-Murphy and Shatwell, 1993).

Mechanical plots were obtained by means of frequency sweep tests performed at a strain value below the critical strain γc, in the LVE zone (2% for all samples). Measurements of the viscoelastic moduli G’ and G” were registered with a range of oscillation frequencies at a constant oscillation amplitude. Figure 5B shows how the viscous (G”) and the elastic (G’) moduli vary with frequency. The storage modulus G’ was higher than the loss modulus G”. This reflects the existence of three-dimensional networks similar to those of strong gels. Thus, in the LVE region, the sample shows solid-like properties. The mechanical plots are representative of hydrogel properties and classification. The hydrogels can be classified as “strong” hydrogels when G’ > G” showing linear viscoelasticity at high strains. “Weak” hydrogels exhibit G′ > G″ linear viscoelasticity just at low strain values at all the detected frequencies (Lapasin and Pricl, 1995). Consequently, the GE-CH hydrogel under study can be considered as a strong gel because of the slight dependence of G’ and G” on the frequency. The data presented here are similar to those in a study by Martínez-Ruvalcaba et al. (2007) on chitosan/xanthan hydrogels. According to the theory of weak gels (Bohlin, 1980), the assessment of the viscoelastic behavior of hydrocolloid gel allows the quantification of the intensity of colloidal forces acting within the polymer network and the interactions among components that interact with each other to a certain extent, forming a single strand. Therefore, the relationship between the mesostructure of a hydrogel and its rheological behavior can be established. The Bohlin coordination number *z* quantifies the number of flow units interacting with one another to give the observed flow response of the material:

(3) $$\begin{array}{c} {\text{G}}^{\prime }(\omega )\sim \omega^{\frac{1}{z}} \end{array}$$

By processing the G’ (w) data of the GE-CH hydrogel, the *z* value was equal to 25.6, confirming the status of the robust structured gel network.

Traditional hydrogels are characterized by weak properties if subjected to a mechanical stimulus or stress. Compared to other hydrogels presented in the literature, the growth of the viscoelastic behavior in response to deformation of the produced GE-CH hydrogel extends the plausible application of the hybrid polymer to different tissue engineering or biomedical applications. In particular, the self-healing properties of hydrogels have an increased value for both 3D bioprinting procedures and injectable systems (Taylor and in het Panhuis, 2016). Therefore, the self-healing properties of the hydrogel were investigated by the application of 800% oscillatory shear strain. After shear removal, the restoration of the dynamic moduli was followed in real time (g 2%, ω 1 Hz). The healing efficiency (HE) was calculated according to Equation 3. A value of HE closer to one indicates the more desirable self-healing capability, whereas a value closer to zero indicates less efficient self-healing (the result is shown in Figure 5C). A completely destructured network and transformation into a liquid-like material (G”>G’) are the immediate results of high shear strain (*g* = 800% for 3 min, ω 1 Hz). Right after cessation of destructive strain, the sample exhibited solid gel responses, with values of instantly restored G′ of around 90% of the original value, with a calculated HE index equal to 0.89. The healed hydrogels were strong enough to sustain repeated stretching; indeed, upon repeating this change of amplitude force, the structure of the hydrogel did not change significantly from that after the first step, and the same healing efficiency (HE=0.88) was obtained. Generally, the self-healing ability of the hydrogel and the time required for the healing process increase with the growth of the viscoelastic behavior in response to deformation of the hydrogels. The observed self-healing properties of the hydrogel may be related to the physical interactions between the amino groups of chitosan and the carboxylic groups of gelatin.

### 3D Spheroid Cultures and 3D Bioprinting

To test the biocompatibility of GE-CH hybrid hydrogel and to investigate the range of potential applications, 3D cell spheroid screening and bioprinted protocols were investigated.

The development of a new 3D *in vitro* model is today an ambitious aim. In the case of biomaterials, embedded and bioprinted models are of great interest to better support cell distribution and cross-talk with the surrounding matrix (as in the tumor microenvironment) so as to investigate drug distribution and the matrix effect around cell aggregates (Fetah et al., 2019; Sun et al., 2020). To set up the experimental conditions for 3D embedded and bioprinted cells, U87 human primary glioblastoma cell lines were selected. Spheroids have gained increasing attention in cell biology studies due to their 3D organization being closer to that of the real cell microenvironment in tissues (Sutherland et al., 1981). Nowadays, the employment of spheroids or more complex organoids is an area of interest for the study of cell biology mechanisms in pathologies, for drug screening purposes, or for tissue engineering and regenerative medicine studies. Today, several commercial materials are under investigation to better control the integrity of spheroid, to study the effect of the microenvironment, and to build up complex tissue-like studies. The viability of spheroids embedded in synthetic matrices or polymers has, in any cases, some limitations. Natural materials can induce undesirable phenomena, like cell migration (Liu et al., 2018), or in certain cases, the selected materials can be toxic or not appropriate for cell-adhesion purposes. The advantages of the employment of biomaterials in cells and spheroid encapsulation are the stability of the construct over time and the compositions of the employed biomaterials (Mironov et al., 2009). The GE-CH crosslinked biomaterial was employed as a hydrogel to encapsulate and maintain U87 3D spheroids. The spheroids were cultured from U87 cells, and, once uniform spheroidal structures (diameter ranging from about 400 to 500 μm) had been obtained, the GE-CH hydrogel was employed to embed the 3D spheroids and to follow their integrity and viability over time.

**Figure 6:**
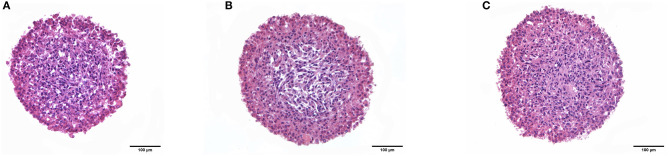
Histological images of spheroids embedded in hydrogel at days 1 **(A)**, 3 **(B)**, and 6 **(C)**.

The results of histological analysis of hydrogel-embedded spheroids are reported in Figure 6 (Figure S2). As shown by staining with H&E, cells in the external layer exhibit a polygonal morphology and tend to be tightly packed, while, in the center of the spheroid, U87 cells are fusiform and less densely packed. However, in all of the observed days, cells appear viable and proliferative and do not show signs of sufferance or degeneration. An interesting observation for the produced hydrogel is related to its ability to maintain the spheroid dimensions and structure, maintaining viability over the culture time points, whereas many of the commercially available matrices result in increased cell migration (Thakuri et al., 2018).

3D bioprinting is a promising fabrication technique, not just for traditional tissue engineering applications but also to model pathological tissues using multiple bioinks and architecture in the third dimension (Gungor-Ozkerim et al., 2018; Sun et al., 2020). The 3D bioprinting process includes cells and polymers in the same “environment.” As a consequence, the properties of the polymers and the crosslinking methodologies employed during the bioprinting process must be biocompatible and easy to tailor. Examples to date include the use of “ionic crosslinkers” (i.e., calcium or divalent salts) or UV and photo-initiator-assisted reactions (i.e., methacrylation, acrylation; Derakhshanfar et al., 2018; Gungor-Ozkerim et al., 2018; Anil Kumar et al., 2019; Ding et al., 2019; Petta et al., 2020; Schipani et al., 2020; Sun et al., 2020). Click reactions can be conducted without the presence of catalyzers or components that can have a detrimental effect; this is advantageous for bioprinting protocols but can be challenging when heterogeneous polymers are employed. The Diels-Alder reaction has already been successfully employed to fabricate 3D bioprintable alginate biopolymers (Ooi et al., 2018). Here, we demonstrate that the same procedure can be adapted to hybrid systems of chitosan and gelatin to expand the employment of the methods to cell populations needed of adhesion motifs.

The bioprinting protocol was assessed, adapting the printing condition of the hydrogel to cell culture. Human U87 glioblastoma cells were employed to preliminarily test the applicability of the produced hydrogel also in bioprinting procedures. In detail, the hybrid hydrogel was dissolved in PBS, and the time of hydrogel network reaction was reduced from 3 to 2.5 h to produce a partially crosslinked material in order to facilitate extrusion in the presence of cells and to obtain homogenous cell-hydrogel solutions.

**Figure 7:**
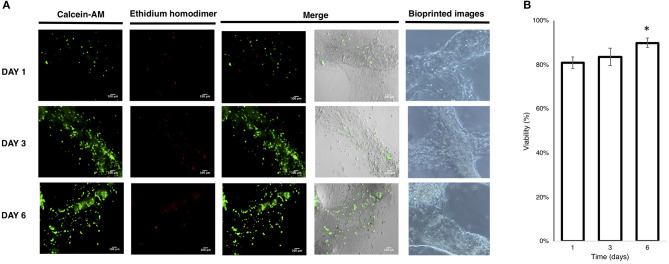
**(A)** Live/Dead imaging of bioprinted U87 cells; **(B)** viability of bioprinted cells. (Mean ± *SD* One-way ANOVA, *n* = 3: *p < 0.005).

The printability was tested operating under sterile conditions on a single Petri dish (35 mm diameter) to avoid contamination and to control the printability and medium change better. The bioprinting process (Video S1) resulted in a printed construct stable in the medium until 6 days of culture, showing the stability of crosslinked cell-laden construct without the employment of additives or salts/crosslinkers to improve the hydrogel shape during the culture media changes. The LIVE/DEAD™ Viability/Cytotoxicity assay (Madl et al., 2016; Zhang et al., 2018) revealed acceptable viability of the bioprinted cells at day 1 and that this increased at days 3 and 6, indicating proliferation of the cells embedded in the hydrogel, as also confirmed by the imaging results (Figures 7A,B).

## Conclusions

In this article, we reported the functionalization of gelatin and chitosan with methyl furan. The polymers obtained were employed with a PEG-star-MA to control the polymerization of hybrid polymers. Network formation by Diels-Alder reaction was preliminarily employed to test the performance of the obtained hydrogels for spheroid encapsulation and 3D bioprinting with the U87 cell line. The hybrid biomaterial obtained was characterized in terms of physicochemical and biological properties. It showed interesting rheological properties, including self-healing features and promising preliminary evidence for biocompatibility. Furthermore, the possibility of employing the GE-CH hydrogel in 3D bioprinting applications opens the way to more detailed studies in the field of tissue engineering and 3D culture for advanced biological studies.

## Data Availability Statement

All datasets generated for this study are included in the article/Supplementary Material.

## Author Contributions

All of the authors contributed to the implementation of the research, to the analysis of the results, and to the writing of the manuscript.

## Conflict of Interest

The authors declare that the research was conducted in the absence of any commercial or financial relationships that could be construed as a potential conflict of interest.

## Supplementary Material

Video S1 Bioprinting process of cell-laden gelatin-chitosan hybrid hydrogel in a grid pattern.

## References

[B1] Afewerki S. Sheikhi A. Kannan S. Ahadian S. Khademhosseini A. (2019). Gelatin-polysaccharide composite scaffolds for 3D cell culture and tissue engineering: towards natural therapeutics. Bioeng. Transl. Med. 4, 96–115. 10.1002/btm2.10124 30680322 PMC6336672

[B2] Alge D. L. Azagarsamy M. A. Donohue D. F. Anseth K. S. (2013). Synthetically tractable click hydrogels for three-dimensional cell culture formed using tetrazine-norbornene chemistry. Biomacromolecules 14, 949–953. 10.1021/bm4000508 23448682 PMC3623454

[B3] Alizadeh M. Abbasi F. Khoshfetrat A. B. Ghaleh H. (2013). Microstructure and characteristic properties of gelatin/chitosan scaffold prepared by a combined freeze-drying/leaching method. Mater. Sci. Eng. C. 33, 3958–3967. 10.1016/j.msec.2013.05.039 23910302

[B4] Anil Kumar S. Alonzo M. Allen S. C. Abelseth L. Thakur V. Akimoto J.. (2019). A visible light-cross-linkable, fibrin-gelatin-based bioprinted construct with human cardiomyocytes and fibroblasts. ACS Biomater. Sci. Eng. 5, 4551–4563. 10.1021/acsbiomaterials.9b00505 32258387 PMC7117097

[B5] Ashammakhi N. Ahadian S. Xu C. Montazerian H. Ko H. Nasiri R.. (2019). Bioinks and bioprinting technologies to make heterogeneous and biomimetic tissue constructs. Mater. Today Bio. 1:100008. 10.1016/j.mtbio.2019.100008 32159140 PMC7061634

[B6] Azagarsamy M. A. Anseth K. S. (2013). Bioorthogonal click chemistry: an indispensable tool to create multifaceted cell culture scaffolds. ACS Macro Lett. 2, 5–9. 10.1021/mz300585q 23336091 PMC3547663

[B7] Bagher Z. Atoufi Z. Alizadeh R. Farhadi M. Zarrintaj P. Moroni L.. (2019). Conductive hydrogel based on chitosan-aniline pentamer/gelatin/agarose significantly promoted motor neuron-like cells differentiation of human olfactory ecto-mesenchymal stem cells. Mater. Sci. Eng. C. Mater. Biol. Appl. 101, 243–253. 10.1016/j.msec.2019.03.068 31029317

[B8] Bohlin L. (1980). A theory of flow as a cooperative phenomenon. J. Colloid Interface Sci. 74, 423–434. 10.1016/0021-9797(80)90211-8

[B9] Carvalho I. C. (2017). Engineered 3D-scaffolds of photocrosslinked chitosan-gelatin hydrogel hybrids for chronic wound dressings and regeneration. Mater. Sci. Eng. C 78, 690–705. 10.1016/j.msec.2017.04.126 28576040

[B10] Cassimjee H. Kumar P. Choonara Y. E. Pillay V. (2020). Proteosaccharide combinations for tissue engineering applications. Carbohydr. Polym. 235:115932. 10.1016/j.carbpol.2020.115932 32122476

[B11] Davidenko N. Schuster C. F. Bax D. V. Farndale R. W. Hamaia S. Best S. M.. (2016). Evaluation of cell binding to collagen and gelatin: a study of the effect of 2D and 3D architecture and surface chemistry. J. Mater. Sci. Mater. Med. 27:148. 10.1007/s10856-016-5763-9 27582068 PMC5007264

[B12] Derakhshanfar S. Mbeleck R. Xu K. Zhang X. Zhong W. Xing M. (2018). 3D bioprinting for biomedical devices and tissue engineering: a review of recent trends and advances. Bioact. Mater. 3, 144–156. 10.1016/j.bioactmat.2017.11.008 29744452 PMC5935777

[B13] Ding H. Illsley N. P. Chang R. C. (2019). 3D bioprinted GelMA based models for the study of trophoblast cell invasion. Sci. Rep. 9:18854. 10.1038/s41598-019-55052-7 31827129 PMC6906490

[B14] Fasolino I. Raucci M. G. Soriente A. Demitri C. Madaghiele M. Sannino A.. (2019). Osteoinductive and anti-inflammatory properties of chitosan-based scaffolds for bone regeneration. Mater. Sci. Eng. C. Mater. Biol. Appl. 105:110046. 10.1016/j.msec.2019.110046 31546343

[B15] Fernandez-Megia E. Novoa-Carballal R. Quiñoá E. Riguera R. (2005). Optimal routine conditions for the determination of the degree of acetylation of chitosan by 1H-NMR. Carbohydr. Polym. 61, 155–161. 10.1016/j.carbpol.2005.04.006

[B16] Fetah K. Tebon P. Goudie M. J. Eichenbaum J. Ren L. Barros N. (2019). The emergence of 3D bioprinting in organ-on-chip systems. Prog. Biomed. Eng. 1:012001 10.1088/2516-1091/ab23df

[B17] Gandavarapu N. R. Azagarsamy M. A. Anseth K. S. (2014). Photo-click living strategy for controlled, reversible exchange of biochemical ligands. Adv. Mater. 26, 2521–2526. 10.1002/adma.201304847 24523204 PMC4528616

[B18] Gandini A. (2013). The furan/maleimide Diels–Alder reaction: a versatile click–unclick tool in macromolecular synthesis. Prog. Polym. Sci. 38, 1–29. 10.1016/j.progpolymsci.2012.04.002

[B19] Gorgieva S. Kokol V. (2012). Preparation, characterization, and *in vitro* enzymatic degradation of chitosan-gelatine hydrogel scaffolds as potential biomaterials. J. Biomed. Mater. Res. A. 100, 1655–1667. 10.1002/jbm.a.34106 22447615

[B20] Gregoritza M. Brandl F. P. (2015). The Diels-Alder reaction: a powerful tool for the design of drug delivery systems and biomaterials. Eur. J. Pharm. Biopharm. 97, 438–453. 10.1016/j.ejpb.2015.06.007 26614562

[B21] Guaresti O. García–astrain C. Urbina L. Eceiza A. Gabilondo N. (2019). Reversible swelling behaviour of diels–alder clicked chitosan hydrogels in response to pH changes. Express Polym. Lett. 13, 27–36. 10.3144/expresspolymlett.2019.4

[B22] Gungor-Ozkerim P. S. Inci I. Zhang Y. S. Khademhosseini A. Dokmeci M. R. (2018). Bioinks for 3D bioprinting: an overview. Biomater. Sci. 6, 915–946. 10.1039/C7BM00765E 29492503 PMC6439477

[B23] Hall D. J. Van Den Berghe H. M. Dove A. P. (2011). Synthesis and post-polymerization modification of maleimide-containing polymers by “thiol-ene” click and Diels–Alder chemistries. Polym. Int. 60, 1149–1157. 10.1002/pi.3121

[B24] Hoch E. Hirth T. Tovar G. E. M. Borchers K. (2013). Chemical tailoring of gelatin to adjust its chemical and physical properties for functional bioprinting. J. Mater. Chem. B 1, 5675–5685. 10.1039/c3tb20745e 32261191

[B25] Huang X. Zhang Y. Zhang X. Xu L. Chen X. Wei S. (2013). Influence of radiation crosslinked carboxymethyl-chitosan/gelatin hydrogel on cutaneous wound healing. Mater. Sci. Eng. C 33, 4816–4824. 10.1016/j.msec.2013.07.044 24094192

[B26] Huynh C. T. Liu F. Cheng Y. Coughlin K. A. Alsberg E. (2018). Thiol-epoxy “click” chemistry to engineer cytocompatible PEG-based hydrogel for siRNA-mediated osteogenesis of hMSCs. ACS Appl. Mater. Interfaces 10, 25936–25942. 10.1021/acsami.8b07167 29986132 PMC6930143

[B27] Irmak G. Demirtaş T. T. Gumusderelioglu M. (2019). Highly methacrylated gelatin bioink for bone tissue engineering. ACS Biomater. Sci. Eng. 5, 831–845. 10.1021/acsbiomaterials.8b00778 33405843

[B28] Jiankang H. Dichen L. Yaxiong L. Bo Y. Hanxiang Z. Qin L.. (2009). Preparation of chitosan-gelatin hybrid scaffolds with well-organized microstructures for hepatic tissue engineering. Acta Biomater. 5, 453–461. 10.1016/j.actbio.2008.07.002 18675601

[B29] Kaur G. Singh G. Singh J. (2018). Photochemical tuning of materials: a click chemistry perspective. Mater. Today Chem. 8, 56–84. 10.1016/j.mtchem.2018.03.002

[B30] Kim E. H. Lim S. Kim T. E. Jeon I. O. Choi Y. S. (2018). Preparation of *in situ* injectable chitosan/gelatin hydrogel using an acid-tolerant tyrosinase. Biotechnol. Bioprocess Eng. 23, 500–506. 10.1007/s12257-018-0315-4

[B31] Koehler K. C. Alge D. L. Anseth K. S. Bowman C. N. (2013a). A diels-alder modulated approach to control and sustain the release of dexamethasone and induce osteogenic differentiation of human mesenchymal stem cells. Biomaterials 34, 4150–4158. 10.1016/j.biomaterials.2013.02.020 23465826 PMC3604741

[B32] Koehler K. C. Anseth K. S. Bowman C. N. (2013b). Diels–Alder mediated controlled release from a poly(ethylene glycol) based hydrogel. Biomacromolecules 14, 538–547. 10.1021/bm301789d 23311608

[B33] Koehler K. C. Anseth K. S. Bowman C. N. Nimmo C. M. Owen S. C. Shoichet M. S.. (2013c). Cross-Linked hydrogels formed through Diels–Alder coupling of furan- and maleimide-modified poly(methyl vinyl ether-alt-maleic acid). Biomacromolecules 14, 1–29. 23157442

[B34] Lapasin R. Pricl S. (1995). Rheology in Rheology of Industrial Polysaccharides: Theory and Applications (Boston, MA: Springer), 162–249. 10.1007/978-1-4615-2185-3_3

[B35] Li Z. He G. Hua J. Wu M. Guo W. Gong J.. (2017). Preparation of γ-PGA hydrogels and swelling behaviors in salt solutions with different ionic valence numbers. RSC Adv. 7, 11085–11093. 10.1039/C6RA26419K30966148

[B36] Lin F. Yu J. Tang W. Zheng J. Defante A. Guo K.. (2013). Peptide-functionalized oxime hydrogels with tunable mechanical properties and gelation behavior. Biomacromolecules 14, 3749–3758. 10.1021/bm401133r 24050500 PMC3871203

[B37] Liu C. Lewin Mejia D. Chiang B. Luker K. E. Luker G. D. (2018). Hybrid collagen alginate hydrogel as a platform for 3D tumor spheroid invasion. Acta Biomater. 75, 213–225. 10.1016/j.actbio.2018.06.003 29879553 PMC6119473

[B38] Liu J. C. Heilshorn S. C. Tirrell D. A. (2004). Comparative cell response to artificial extracellular matrix proteins containing the RGD and CS5 cell-binding domains. Biomacromolecules 5, 497–504. 10.1021/bm034340z 15003012

[B39] Ma T. Gao X. Dong H. He H. Cao X. (2017). High-throughput generation of hyaluronic acid microgels via microfluidics-assisted enzymatic crosslinking and/or Diels–Alder click chemistry for cell encapsulation and delivery. Appl. Mater. Today 9, 49–59. 10.1016/j.apmt.2017.01.00732362581

[B40] Madl C. M. Heilshorn S. C. (2019). Rapid Diels–Alder cross-linking of cell encapsulating hydrogels. Chem. Mater. 31, 8035–8043. 10.1021/acs.chemmater.9b02485 32410775 PMC7224313

[B41] Madl C. M. Katz L. M. Heilshorn S. C. (2016). Bio-orthogonally crosslinked, engineered protein hydrogels with tunable mechanics and biochemistry for cell encapsulation. Adv. Funct. Mater. 26, 3612–3620. 10.1002/adfm.201505329 27642274 PMC5019573

[B42] Mao J. Kondu S. Ji H. F. McShane M. J. (2006). Study of the near-neutral pH-sensitivity of chitosan/gelatin hydrogels by turbidimetry and microcantilever deflection. Biotechnol. Bioeng. 95, 333–441. 10.1002/bit.20755 16894636 PMC4465413

[B43] Martínez-Ruvalcaba A. Chornet E. Rodrigue D. (2007). Viscoelastic properties of dispersed chitosan/xanthan hydrogels. Carbohydr. Polym. 67, 586–595. 10.1016/j.carbpol.2006.06.033

[B44] Mironov V. Visconti R. P. Kasyanov V. Forgacs G. Drake C. J. Markwald R. R. (2009). Organ printing: tissue spheroids as building blocks. Biomaterials 30, 2164–2174. 10.1016/j.biomaterials.2008.12.084 19176247 PMC3773699

[B45] Moroni L. Burdick J. A. Highley C. Lee S. J. Morimoto Y. Takeuchi S.. (2018). Biofabrication strategies for 3D *in vitro* models and regenerative medicine. Nat. Rev. Mater. 3, 21–37. 10.1038/s41578-018-0006-y 31223488 PMC6586020

[B46] Nair D. P. Podgórski M. Chatani S. Gong T. Xi W. Fenoli C. R. (2014). The Thiol-Michael addition click reaction: a powerful and widely used tool in materials chemistry. Chem. Mater. 26, 724–744. 10.1021/cm402180t

[B47] Neves S. C. Moroni L. Barrias C. C. Granja P. L. (2019). Leveling up hydrogels: hybrid systems in tissue engineering. Trends Biotechnol. 38, 292–315. 10.1016/j.tibtech.2019.09.004 31787346

[B48] Nicolas J. Magli S. Rabbachin L. Sampaolesi S. Nicotra F. Russo L. (2020). 3D extracellular matrix mimics: fundamental concepts and role of materials chemistry to influence the stem cell fate. Biomacromolecules 21, 1968–1994. 10.1021/acs.biomac.0c00045 32227919

[B49] Nimmo C. M. Owen S. C. Shoichet M. S. (2011). Diels–Alder click cross-linked hyaluronic acid hydrogels for tissue engineering. Biomacromolecules 12, 824–830. 10.1021/bm101446k 21314111

[B50] Nimmo C. M. Shoichet M. S. (2011). Regenerative biomaterials that “click”: simple, aqueous-based protocols for hydrogel synthesis, surface immobilization, and 3D patterning. Bioconjug. Chem. 22, 2199–2209. 10.1021/bc200281k 21995458

[B51] Ooi H. W. Hafeez S. van Blitterswijk C. A. Moroni L. Baker M. B. (2017). Hydrogels that listen to cells: a review of cell-responsive strategies in biomaterial design for tissue regeneration. Mater. Horizons 4, 1020–1040. 10.1039/C7MH00373K

[B52] Ooi H. W. Mota C. Ten Cate A. T. Calore A. Moroni L. Baker M. B. (2018). Thiol-ene alginate hydrogels as versatile bioinks for bioprinting. Biomacromolecules 19, 3390–3400. 10.1021/acs.biomac.8b00696 29939754 PMC6588269

[B53] Park E. J. Gevrek T. N. Sanyal R. Sanyal A. (2014). Indispensable platforms for bioimmobilization: maleimide-based thiol reactive hydrogels. Bioconjug. Chem. 25, 2004–2011. 10.1021/bc500375r 25250772

[B54] Petta D. D'Amora U. Ambrosio L. Grijpma D. Eglin D. D'Este M. (2020). Hyaluronic acid as a (bio)ink for extrusion-based 3D printing. Biofabrication. 12:032001. 10.1088/1758-5090/ab8752 32259809

[B55] Polgar L. M. Kingma A. Roelfs M. van Essen M. van Duin M. Picchioni F. (2017). Kinetics of cross-linking and de-cross-linking of EPM rubber with thermoreversible Diels-Alder chemistry. Eur. Polym. J. 90, 150–161. 10.1016/j.eurpolymj.2017.03.020

[B56] Rodríguez-Rodríguez R. Espinosa-Andrews H. Velasquillo-Martínez C. García-Carvajal Z. Y. (2020). Composite hydrogels based on gelatin, chitosan and polyvinyl alcohol to biomedical applications: a review. Int. J. Polym. Mater. Polym. Biomater. 69, 1–20. 10.1080/00914037.2019.1581780

[B57] Ross-Murphy S. B. Shatwell K. P. (1993). Polysaccharide strong and weak gels. Biorheology 30, 217–227. 10.3233/BIR-1993-303-407 8286724

[B58] Roy N. Bruchmann B. Lehn J. M. (2015). DYNAMERS: Dynamic polymers as self-healing materials. Chem. Soc. Rev. 44, 3786–3807. 10.1039/C5CS00194C 25940832

[B59] Ruprai H. Romanazzo S. Ireland J. Kilian K. Mawad D. George L.. (2019). Porous chitosan films support stem cells and facilitate sutureless tissue repair. ACS Appl. Mater. Interfaces 11, 32613–32622. 10.1021/acsami.9b09123 31418544

[B60] Russo L. Battocchio C. Secchi V. Magnano E. Nappini S. Taraballi F.. (2014). Thiol-ene mediated neoglycosylation of collagen patches: a preliminary study. Langmuir 30, 1336–1342. 10.1021/la404310p 24443819

[B61] Russo L. Sgambato A. Visone R. Occhetta P. Moretti M. Rasponi M. (2016). Gelatin hydrogels via thiol-ene chemistry. Monatshefte fur Chemie 147:5 10.1007/s00706-015-1614-5

[B62] Russo L. Zanini S. Riccardi C. Nicotra F. Cipolla L. (2011). Diazo transfer for azido-functional surfaces. Mater. Today 14:8 10.1016/S1369-7021(11)70088-8

[B63] Saraiva S. M. Miguel S. P. Ribeiro M. P. Coutinho P. Correia I. J. (2015). Synthesis and characterization of a photocrosslinkable chitosan-gelatin hydrogel aimed for tissue regeneration. RSC Adv. 5, 63478–63488. 10.1039/C5RA10638A

[B64] Schindelin J. Arganda-Carreras I. Frise E. Kaynig V. Longair M. Pietzsch T.. (2012). Fiji: an open-source platform for biological-image analysis. Nat. Methods 9, 676–682. 10.1038/nmeth.2019 22743772 PMC3855844

[B65] Schipani R. Scheurer S. Florentin R. Critchley S. E. Kelly D. J. (2020). Reinforcing interpenetrating network hydrogels with 3D printed polymer networks to engineer cartilage mimetic composites. Biofabrication. 12:035011. 10.1088/1758-5090/ab8708 32252045

[B66] Smith L. J. Taimoory S. M. Tam R. Y. Baker A. E. G. Binth Mohammad N. Trant J. F.. (2018). Diels-Alder click-cross-linked hydrogels with increased reactivity enable 3D cell encapsulation. Biomacromolecules 19, 926–935. 10.1021/acs.biomac.7b01715 29443512

[B67] Spicer C. D. Pashuck E. T. Stevens M. M. (2018). Achieving controlled biomolecule-biomaterial conjugation. Chem. Rev. 118, 7702–7743. 10.1021/acs.chemrev.8b00253 30040387 PMC6107854

[B68] Stewart S. A. Backholm M. Burke N. A. D. Stöver H. D. H. (2016). Cross-linked hydrogels formed through diels–alder coupling of furan- and maleimide-modified poly(methyl vinyl ether-alt-maleic acid). Langmuir 32, 1863–1870. 10.1021/acs.langmuir.5b04450 26800849

[B69] Sultankulov B. Berillo D. Sultankulova K. Tokay T. Saparov A. (2019). Progress in the development of chitosan-based biomaterials for tissue engineering and regenerative medicine. Biomolecules 9:470. 10.3390/biom9090470 31509976 PMC6770583

[B70] Sun W. Starly B. Daly A. C. Burdick J. A. Groll J. Skeldon G.. (2020). The bioprinting roadmap. Biofabrication 12:22002. 10.1088/1758-5090/ab5158 32031083

[B71] Sutherland R. Carlsson J. Durand R. Yuhas J. (1981). Spheroids in cancer research. Cancer Res. 41, 2980–2984.

[B72] Tam R. Y. Smith L. J. Shoichet M. S. (2017). Engineering cellular microenvironments with photo- and enzymatically responsive hydrogels: toward biomimetic 3D cell culture models. Acc. Chem. Res. 50, 703–713. 10.1021/acs.accounts.6b00543 28345876

[B73] Tao F. Cheng Y. Shi X. Zheng H. Du Y. Xiang W.. (2020). Applications of chitin and chitosan nanofibers in bone regenerative engineering. Carbohydr. Polym. 230:115658. 10.1016/j.carbpol.2019.115658 31887899

[B74] Taraballi F. Russo L. Battocchio C. Polzonetti G. Nicotra F. Cipolla L. (2014). A model study for tethering of (bio)active molecules to biomaterial surfaces through arginine. Org. Biomol. Chem. 12, 4089–92. 10.1039/c4ob00160e 24838600

[B75] Taylor D. L. in het Panhuis M. (2016). Self-healing hydrogels. Adv. Mater. 28, 9060–9093. 10.1002/adma.201601613 27488822

[B76] Thakuri P. S. Liu C. Luker G. D. Tavana H. (2018). Biomaterials-based approaches to tumor spheroid and organoid modeling. Adv. Healthc. Mater. 7:e1700980. 10.1002/adhm.201700980 29205942 PMC5867257

[B77] Varaprasad K. Reddy N. N. Ravindra S. Vimala K. Raju K. M. (2011). Synthesis and characterizations of macroporous poly(acrylamide-2-acrylamido-2-methyl-1-propanesulfonic acid) hydrogels for *in vitro* drug release of ranitidine hydrochloride. Int. J. Polym. Mater. 60, 490–503. 10.1080/00914037.2010.531816

[B78] Wang Y. Pitto-Barry A. Habtemariam A. Romero-Canelon I. Sadler P. J. Barry N. P. E. (2016). Nanoparticles of chitosan conjugated to organo-ruthenium complexes. Inorg. Chem. Front. 3, 1058–1064. 10.1039/C6QI00115G

[B79] Zhang L. G. Fisher J. P. Leong K. W. (2015). 3D Bioprinting and Nanotechnology in Tissue Engineering and Regenerative Medicine. Elvesier Inc. 10.1016/C2013-0-18595-9

[B80] Zhang Y. S. Oklu R. Dokmeci M. R. Khademhosseini A. (2018). Three-dimensional bioprinting strategies for tissue engineering. Cold Spring Harb. Perspect. Med. 8:a025718 10.1101/cshperspect.a025718 28289247 PMC5793742

[B81] Zhao W. Glavas L. Odelius K. Edlund U. Albertsson A.-C. (2014). A robust pathway to electrically conductive hemicellulose hydrogels with high and controllable swelling behavior. Polymer (Guildf) 55, 2967–2976. 10.1016/j.polymer.2014.05.003

