# Supplementary material for: Design and Synthesis of Chitosan—Gelatin Hybrid Hydrogels for 3D Printable in vitro Models
Source: Front Chem. 2020 Jul 14;8:524. doi: 10.3389/fchem.2020.00524 (PMC7373092; doi:10.3389/fchem.2020.00524)
Supplement: Supplementary file 2 [file Data_Sheet_1.pdf]

Supplementary Material

1. SUPPLEMENTARY FIGURES

A)

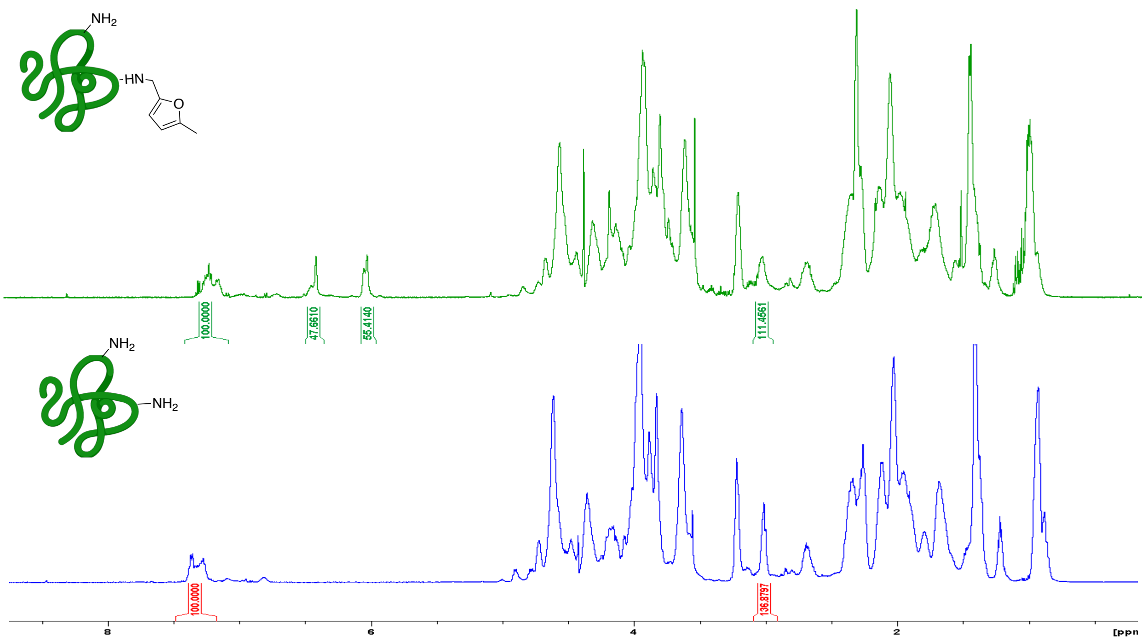

B)

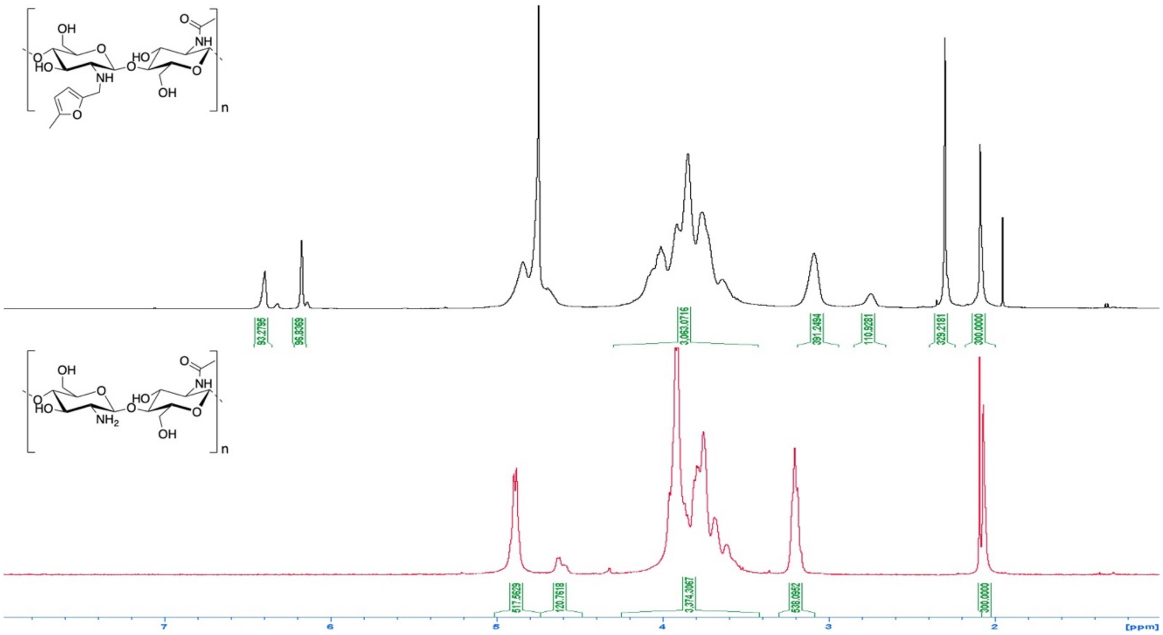

Figure S1: Integrated spectra of gelatin (A) and chitosan (B) unfunctionalized and functionalized with 5-methylfurfural.

A)

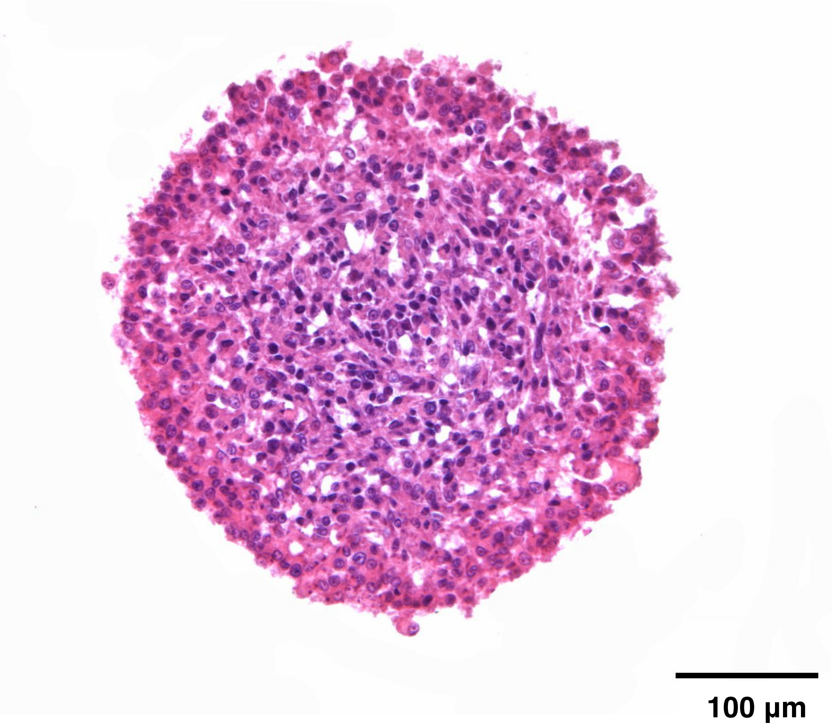

B)

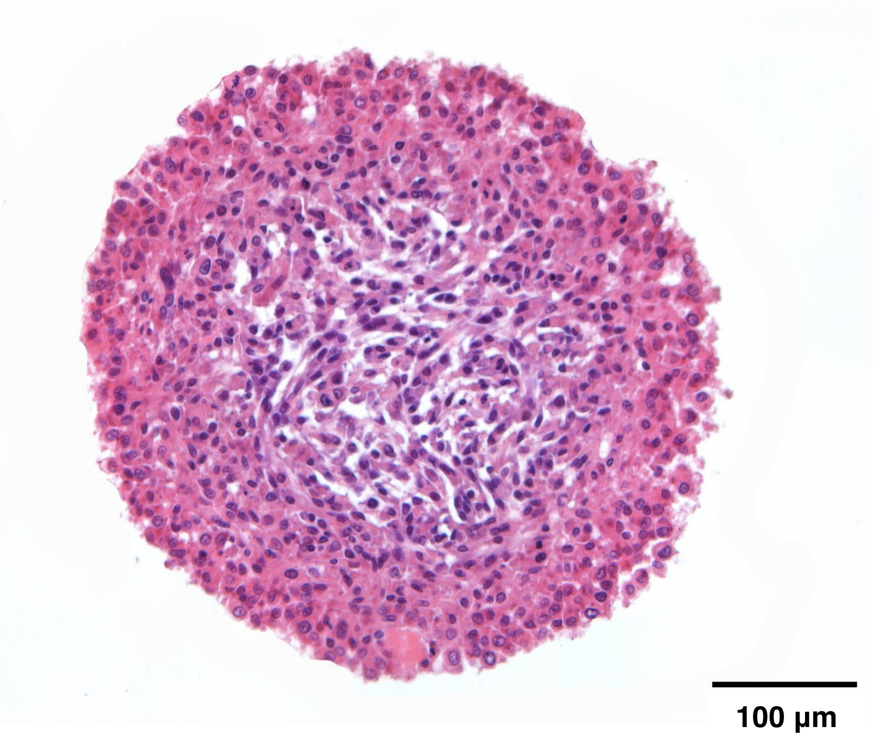

C)

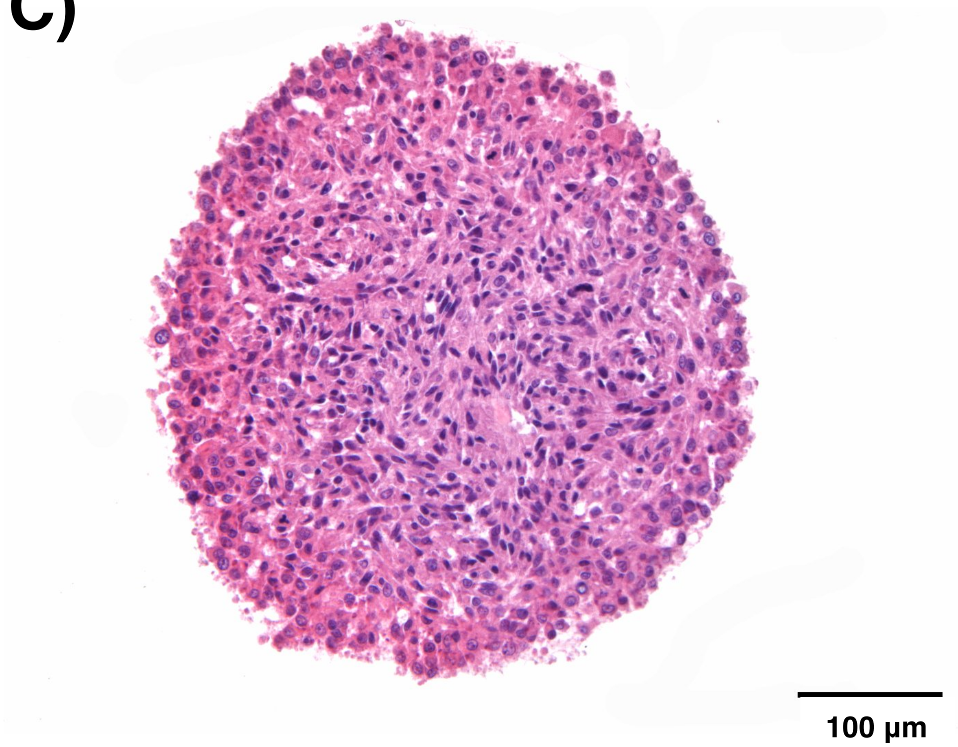

Figure S2. Histological images of spheroids embedded in GE-CH hydrogel at day 1 (A), 3 (B) and 6 (C).
